# COMMUNITY NETWORKS ADDRESSING AGING WITH LIFELONG DISABILITIES

**DOI:** 10.1093/geroni/igz038.2757

**Published:** 2019-11-08

**Authors:** Edward F Ansello

**Affiliations:** Virginia Commonwealth University, Richmond, Virginia, United States

## Abstract

Adults aging with lifelong developmental disabilities (I/DD: Down syndrome and other intellectual disabilities, cerebral palsy, etc.) continue to pose a challenge to the Aging Network, developmental services, and healthcare systems. Some funded projects, model programs, and episodic initiatives have helped advance intersystem collaboration but there is still scant federal or state public policy specific to aging with lifelong disabilities. The Area Planning and Services Committee (APSC), a product of three consecutive AoA grants on building and testing intersystem cooperation/collaboration between these systems, is a partnership of community based organizations and academe operating in metro Richmond since 2003. Its successful hands on, interdisciplinary management process has implemented needs assessments, statewide training, staff development, dementia awareness, etc., and been a model for the Virginia Geriatric Education Center (VGEC) Plenary overseeing all GWEP programs. The APSC history informs VGEC GWEP’s incorporating I/DD content into faculty development programs, microlearning, ECHO, and other practitioner training.

